# A systematic review and meta-analysis of metal versus plastic stents for drainage of pancreatic fluid collections: metal stents are advantageous

**DOI:** 10.1007/s00464-018-6416-5

**Published:** 2018-09-06

**Authors:** Rebecca Saunders, Jayapal Ramesh, Silvia Cicconi, Jonathan Evans, Vincent S. Yip, Michael Raraty, Paula Ghaneh, Robert Sutton, John P. Neoptolemos, Christopher Halloran

**Affiliations:** 1 Pancreatitis Research Group, Royal Liverpool University Hospital, Liverpool, UK; 2 Gastroenterology, Royal Liverpool University Hospital, Liverpool, UK; 3 Liverpool Cancer Trials Unit, University of Liverpool, Liverpool, UK; 4 Radiology, Royal Liverpool University Hospital, Liverpool, UK; 5 HPB Surgery, Barts Healthcare, London, UK; 6 Institute of Translational Medicine, University of Liverpool, Liverpool, UK; 7 General Surgery, Universitatsklinikum Heidelberg Chirurgische Klinik, Heidelberg, Germany

**Keywords:** Pancreatic fluid collection, Metal stents, Plastic stents, Endoscopic ultrasound intervention, Pancreatic pseudocyst, Drainage

## Abstract

**Background:**

The use of fully covered metal stents (FCSEMS) and specifically designed lumen apposing metal stents for transmural drainage of pancreatic fluid collections has become widespread. A systematic review published in 2015 did not support the routine use of metal stents for drainage of pancreatic fluid collections. However, recent studies have shown conflicting data; therefore a systematic review and meta-analysis was performed.

**Method:**

We conducted a database search for original comparative studies between plastic and metal stents. The random effects model was used to calculate pooled risk ratios (RR) with 95% confidence intervals (CI). Outcomes analysed were clinical success, adverse events and requirement of further intervention.

**Results:**

The search identified 936 studies, 7 studies with 681 (340 metal, 341 plastic) patients met inclusion criteria and were included in the meta-analysis. Clinical success was achieved in 93.8% versus 86.2% in the metal and plastic groups, respectively, RR 1.08 [95% CI 1.02–1.14]; *p* = 0.009. Adverse events were reduced for metal stents when compared with plastic (10.2% vs. 25.0%), RR 0.42 [95% CI 0.22–0.81]; *p* = 0.010. Metal stent usage reduced bleeding (2.8% vs. 7.9%), RR 0.37; [95% CI 0.18–0.75]; *p* = 0.006. Further intervention was required in 12.4% of patients in the metal stent group versus 26.7% for plastic stents, RR 0.54; [95% CI 0.22–1.29]; *p* = 0.165.

**Conclusions:**

The use of metal stents for drainage of pancreatic fluid collections is associated with improved clinical success, fewer adverse events and reduced bleeding compared to plastic stents.

**Electronic supplementary material:**

The online version of this article (10.1007/s00464-018-6416-5) contains supplementary material, which is available to authorized users.

Pancreatic and peri-pancreatic fluid collections (PFC) are common following an insult to the pancreas [1, 2]. It is important to differentiate between those, which are purely fluid, and those that contain necrotic tissue when considering appropriate treatments. The revised Atlanta Classification states that acute peri-pancreatic fluid collections (APFC) are homogenous, do not have a well-defined wall and can be multiple. They occur within the first 4 weeks of non-necrotic interstitial oedematous pancreatitis. Most APFC remain sterile and resolve spontaneously without intervention, they do not by themselves constitute severe acute pancreatitis [3]. Pancreatic pseudocysts are peri-pancreatic fluid collections surrounded by a well-defined wall with no solid material and markedly increased amylase activity. A pancreatic pseudocyst usually arises after more than 4 weeks of the start of an attack and are nearly always associated with chronic pancreatitis. A pseudocyst is extremely rare in acute pancreatitis and use of the term pancreatic pseudocyst in the setting of acute pancreatitis may fall into disuse [3]. A pseudocyst may occur in acute necrotising pancreatitis secondary to a disrupted main pancreatic duct, whereby parenchymal necrosis of the neck or body isolates a viable distal remnant [3, 4].

Acute necrotising pancreatitis may feature acute necrotic collections (ANC), which have mixed heterogeneous contents with no definable wall or capsule. Walled-off necrosis (WON), which may be intrapancreatic or extrapancreatic, has mixed fluid and solid components as well as a defined capsule and requires at least 4 weeks following the onset of necrotising pancreatitis to mature [3].

Although many PFCs will resolve spontaneously, intervention is indicated in cases when infection is present or if the collection is persistently symptomatic [3, 5]. Management options for PFCs include percutaneous, endoscopic, minimal access and open surgical techniques [6–8]. A recent randomised trial has shown equal efficacy between surgery and endoscopic drainage of pseudocysts but found reduced length of hospital stay and reduced costs for endoscopic intervention [9]. Thus, endoscopic management is now often regarded as first-line management of PFCs with multiple studies demonstrating its safety and high success rates [10, 11].

Endoscopic drainage of PFCs traditionally involves creating a fistula and placement of plastic stents to enable resolution by transluminal drainage. Natural progression led to the use of fully covered self-expanding metal stents (FCSEMS), initially designed for biliary stenting and latterly specifically designed FCSEMS as well as lumen apposing metal stents (LAMS) [10, 12]. Metal stents have the advantage of large diameter lumens, which facilitate better drainage, particularly when there is debris or necrotic tissue present. They also allow easy and safe access to the cavity for direct endoscopic necrosectomy if required [13]. However, metal stents are significantly more expensive than plastic stents and some early reports raised safety concerns regarding their use, notably delayed bleeding and embedded stents [14]. With high success rates using plastic stents published, some centres do not see the benefit of metal stents, particularly for pseudocyst drainage [10].

A systematic review published in 2015 concluded that there was no evidence to support the routine use of metal stents for drainage of pancreatic fluid collections [15]. Since then, however, several studies comparing plastic double pigtail stents and FCSEMS/LAMS have been published in the literature.

The aim of this systematic review and meta-analysis is to review these recently published studies to assess clinical success rates, adverse events and requirement of further intervention, when treating PFC of any description.

## Materials and methods

### Eligibility criteria

The inclusion criteria for qualitative and quantitative analysis were comparative studies between plastic double pigtail stents and metal stents for drainage of both walled-off necrosis (WON) and pseudocysts. Randomised controlled trials, prospective and retrospective studies were all eligible for inclusion as preliminary searches demonstrated few randomised controlled trials. Studies that used LAMS, FCSEMS and biliary self-expanding metal stents were all included. Only English language adult studies were included. No date criteria were set. The review was conducted according to the Preferred Reporting Items for Systematic Reviews and Meta-analyses (PRISMA) [16] and the protocol was registered on PROSPERO (http://www.crd.york.ac.uk/PROSPERO, CRD42017071101).

### Information sources

MEDLINE, Pubmed and SCOPUS databases were searched, with the final search conducted on 20/10/17. References of included studies were also screened.

### Search

The search terms were “pseudocyst” OR “pancreatic fluid collection” OR “walled-off necrosis” AND “endoscopy” OR “endoscopic ultrasound” OR “EUS” AND “stent”.

### Study selection

Search results were combined on the Covidence software platform. Duplicate records were removed. Two reviewers (RSa, JR) independently scanned the title and abstract of all records identified during the search. Full-text articles were retrieved and reviewed if it was not clear from the abstract if inclusion criteria were met. We included studies irrespective of whether they reported all outcome measures. Studies not meeting the inclusion criteria were excluded with the reason for exclusion recorded.

### Data collection process

Data were extracted independently in a standardised table by two reviewers (RSa, SC). Agreement was reached by consensus.

### Data items

The following characteristics were extracted from the studies: study design, number of centres, location of centres, date of studies, total number of participants, mean age, sex, type of PFC, type of metal stent, type and number of plastic stent, follow-up period and size of PFC.

The primary outcome measure recorded was clinical success, defined as resolution of pancreatic fluid collection. Secondary outcome measures were adverse events and rate of reintervention. Other outcomes recorded were technical success, recurrence, length of stay and stent migration.

### Statistics

Random effects modelling was undertaken for each of the outcomes of interest. The effect size between metal and plastic stents was described in terms of individual and pooled risk ratios with 95% confidence intervals and weighting estimated using the Mantel–Haenszel method. Forest plots were generated and study heterogeneity was investigated using the *I*^2^ statistic. An *I*^2^ exceeding 50% was considered to indicate significant heterogeneity. Sensitivity analyses were performed on the outcomes when heterogeneity or outlier studies were found. The effect size between metal and plastic stents was also explored for pseudocyst and WON separately. Funnel plots were used to explore the presence of publication bias and Egger’s regression test for assessing their asymmetry. We considered *p* values < 0.05 to be statistically significant. All the analyses were performed in Stata 14 (StataCorp, College Station, Texas, USA), using the command *Metan* for fitting random effects models and producing forest plots.

## Results

### Study selection

**Fig. 1:**
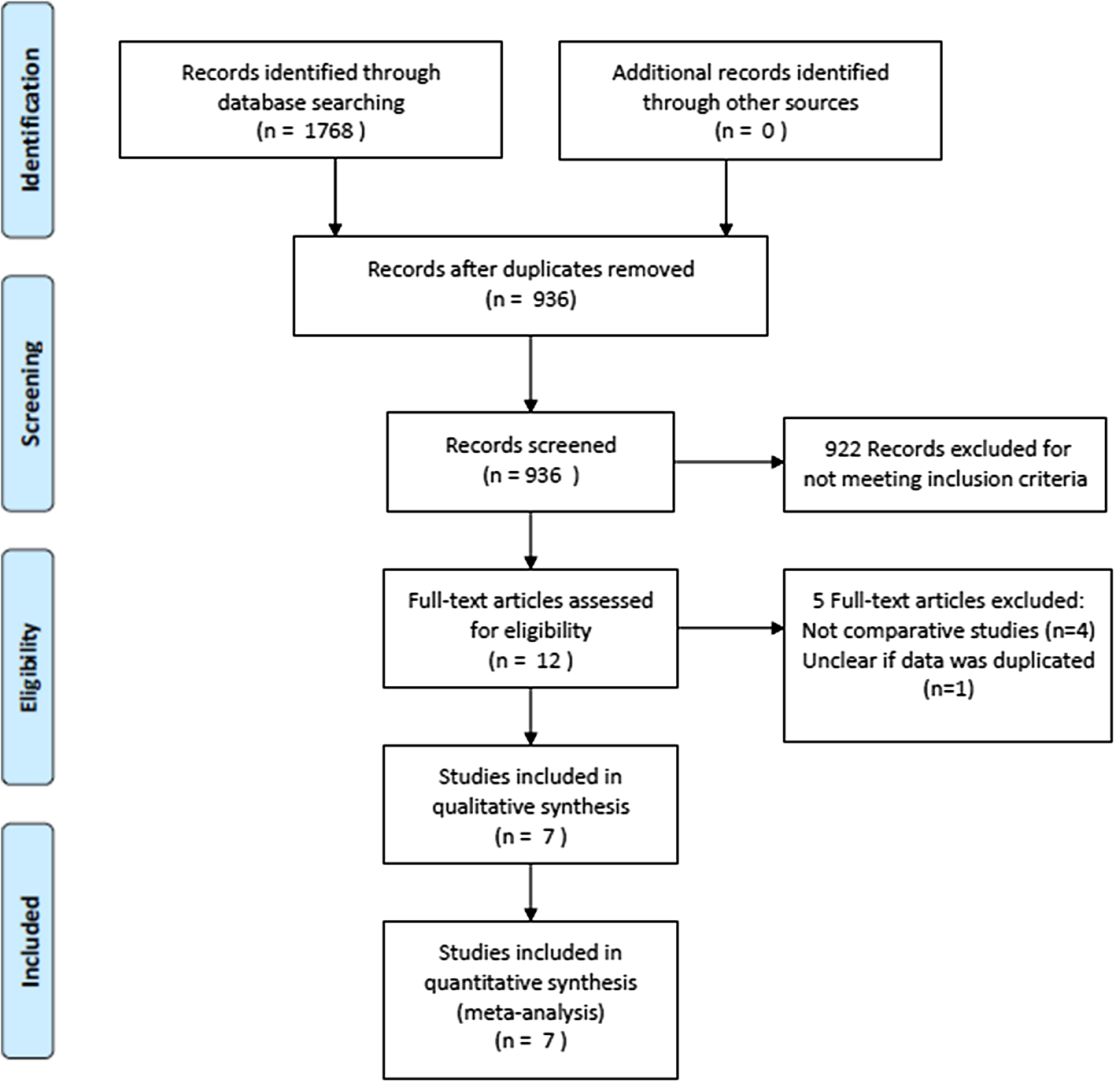
PRISMA flow chart of search [16]

The database search returned 1768 articles, 936 remained after duplicates were removed (see Fig. 1). Twelve full-text articles were reviewed and five were excluded; four were not comparative studies and another was from the same centre as an included study [17, 18] and it was unclear if the data were duplicated. Seven studies were included in the analysis [17, 19–24]. It is important to state that patient allocation to study group was by stent, rather than by type of pancreatic fluid collection.

### Study characteristics

The characteristics of included studies are shown in Table 1. Patient demographic information and characteristics are summarised in Table 2. The outcome measures of individual studies are summarised in Table 3.

**Table 1:** Characteristics of included studies

Author Year	Study type	Number of patients (%)		PFC type (%)		Metal stent type (diameter mm)	Plastic stent size (number of stents)
		Plastic stent	Metal stent	Pseudocyst	WON		
Ang et al. 2016 [24]	Retrospective 2 centre	37 (76)	12 (24)	31 (63)	18 (37)	Nagi (16 mm)	(1–2)
Bang et al. 2016 [19]	Retrospective case control	40 (67)	20 (33)	21 (35)	39 (65)	Hot AXIOS (15 mm)	7f 4 cm (2)
Bapaye et al. 2016 [20]	Retrospective	61 (46)	72 (54)	–	133 (100)	Nagi (16 mm)	7f (2–4)
Dayyeh et al 2017 [23]	Retrospective	36 (38)	58 (62)	–	94 (100)	Axios (15 mm), Niti-s (18 or 20 mm)	7f or 10f (2 or more)
Lee et al. 2014 [22]	*RCT	25 (50)	25 (50)	14 (28)	36 (72)	BONA-Soo (8 mm)	7f (2–3)
Mukai et al. 2014 [21]	Retrospective	27 (39)	43 (61)	–	70 (100)	Axios (10 or 15 mm) Niti-s (16 mm) Hanaro (12 mm)	7f (1–2)
Shariaha et al 2015 [24]	Retrospective 2 centre cohort	118 (51)	112 (49)	230 (100)	–	Wallflex Gore Viabl (10 mm)	10f (2)

*In Lee et al., five patients were lost to follow-up (3 and 2 in plastic and metal stents, respectively). Therefore, the number of patients used for calculating clinical success, reintervention and recurrence was 45 (22/23)

**Table 2:** Patient demographics and characteristics in included studies

Author Year	Mean age, years		Male (%)		Mean PFC size (mm)		PFC infection (%)		Nasocystic drainage (%)		Median follow-up duration (months)	
	Plastic	Metal	Plastic	Metal	Plastic	Metal	Plastic	Metal	Plastic	Metal	Plastic	Metal
Ang et al. 2016 [24]	*Cross-over of stent summary presented		*Cross-over of stent summary presented		*Cross-over of stent summary presented		–	–	Not routine		–	
Bang et al. 2016 [19]	52.9	50.7	62.5	55.0	109.3	120.0	–	–	20.0	5.0	26.5	5.3
Bapaye et al. 2016 [20]	40.7	43.9	88.5	86.1	117.1	100.9	–	–	Yes		Until stent removal	
Dayyeh et al. 2017 [23]	59.7	52.7	77.7	77.6	128.0	134.0	44.4	39.7	No		–	
Lee et al. 2014 [22]	51.6	53.7	76.0	88.0	89.0	84.0	32.0	44.0	If debris/pus		–	
Mukai et al. 2014 [21]	55.9	54.4	77.8	86.0	77.1	105.6	59.3	53.4	92.6	25.6	–	
Sharaiha et al. 2015 [17]	52.2	53.2	69.5	55.4	97.8	98.6	–	–	No		16	

*Ang et al. reports a cross-over summary of patient characteristics. Initial stent placement was plastic in 37 patients and metal in 12, 4 patients with plastic stents went on to have metal stents inserted at a further procedure

**Table 3:** Summary table of outcome measures

Author Year	Technical success (%)		Clinical success (%)		Adverse events (%)		PFC recurrence (%)		Reintervention (%)		Mean length of stay (days)	
	Plastic	Metal	Plastic	Metal	Plastic	Metal	Plastic	Metal	Plastic	Metal	Plastic	Metal
Ang et al. 2016 [24]	100.0	100.0	94.6	100.0	13.5	0.0	–		35.1	8.3	–	
Bang et al. 2016 [19]	100.0	100.0	92.5	95.0	15.0	20.0	0.0	0.0	30.0	25.0	9.2	9.3
Bapaye et al. 2016 [20]	100.0	100.0	73.8	94.4	36.1	5.6	0.0	0.0	26.2	2.8	8.0	4.1
Dayyeh et al 2017 [23]	–		75.0	82.8	Summaries of specific AE presented		–		–		8.0*	4.0*
Lee et al. 2014 [22]	100.0	100.0	90.9	87.0	8.0	0.0	0.0	4.5	9.1	13.0	–	
Mukai et al. 2014 [21]	100.0	100.0	92.6	97.7	18.5	7.0	–		25.9	23.3	28.7	22.5
Sharaiha et al. 2015 [17]	92.0	98.0	89.0	98.2	31.4	16.1	3.4	0.9	–		–	

*Dayyeh et al. summarised median length of stay

### Synthesis of results

#### Clinical success

**Fig. 2:**
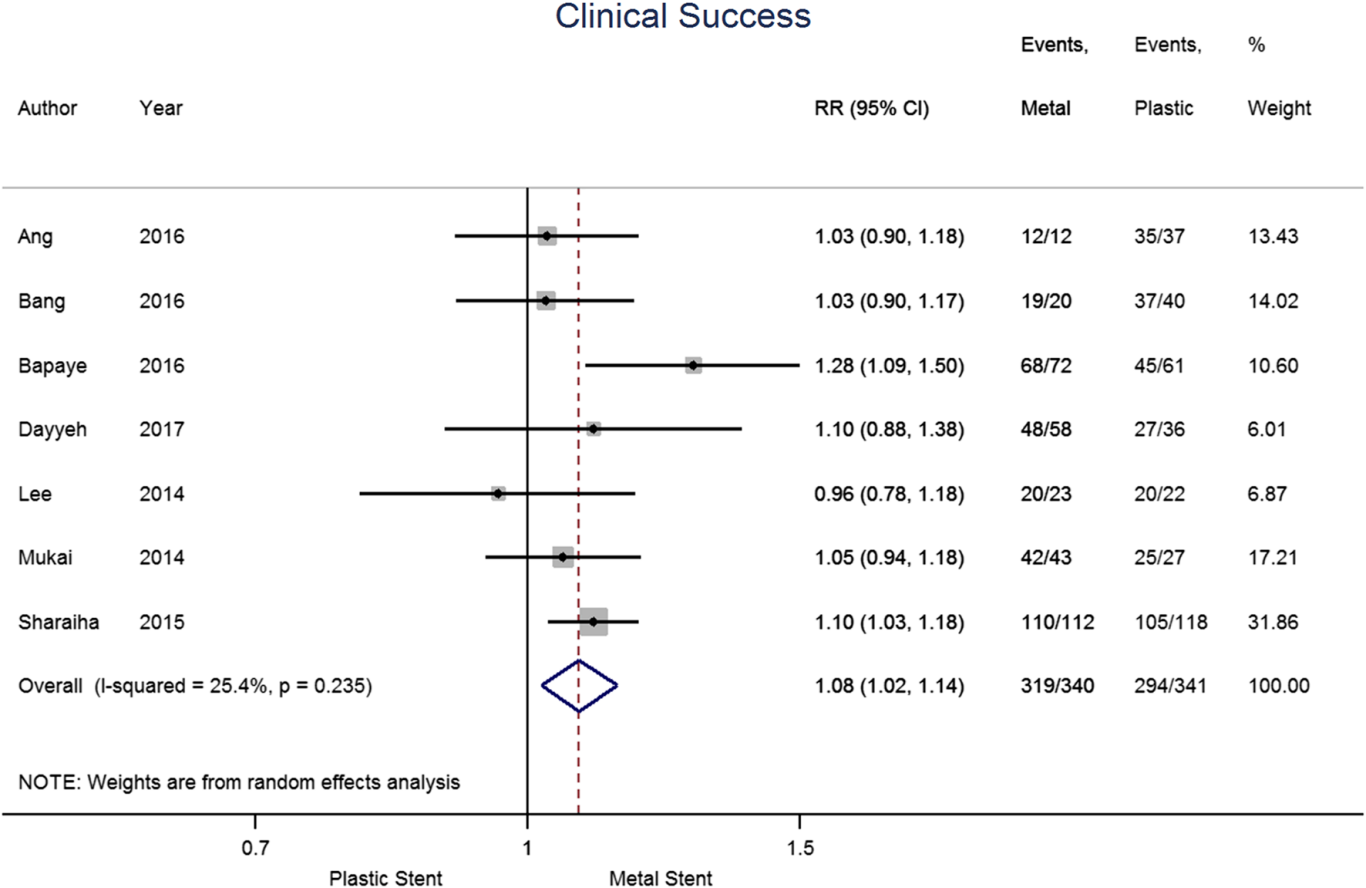
Forest plot for individual and pooled risk ratio of clinical success

The results for the primary outcome measure of clinical success are shown in Fig. 2. The seven papers included in this analysis contained a total of 681 patients, 340 and 341 had metal and plastic stents, respectively. Overall, 93.8% of patients in the metal stent group and 86.2% in the plastic stent group achieved clinical success. The pooled risk ratio (RR) suggests an increase in clinical success when metal stents are used compared to plastic stents (1.08 [95% CI 1.02–1.14], *p* = 0.009; *I*^2^ = 25.4%).

There was heterogeneity of definition of clinical success between studies, summarised in Table 4. Five studies defined success using both radiological and clinical criteria. One study assessed clinical improvement only and one study reported radiological resolution. For the Ang et al., we included final clinical success for the quantitative analysis, for Dayyeh et al., we included the results that regarded concomitant percutaneous drainage as a failure of endoscopic drainage for better consistency across studies.

**Table 4:** Definitions of clinical success

Author Year	Definition clinical success
Ang et al. 2016 [24]	Size < 2 cm on imaging and resolution of symptoms
Bang et al. 2016 [19]	Size < 2 cm on imaging with resolution of symptoms at 8 weeks
Bapaye et al. 2016 [20]	Symptom resolution and complete resolution on imaging at end of treatment period
Dayyeh et al. 2017 [23]	Complete clinical amelioration of acute index symptoms and resolution on imaging
Lee et al. 2014 [22]	Size < 2 cm on CT performed every 4 weeks with resolution of symptoms
Mukai et al. 2014 [21]	Resolution of symptoms
Sharaiha et al. 2015 [17]	Resolution at 12 months on imaging

**Fig. 3:**
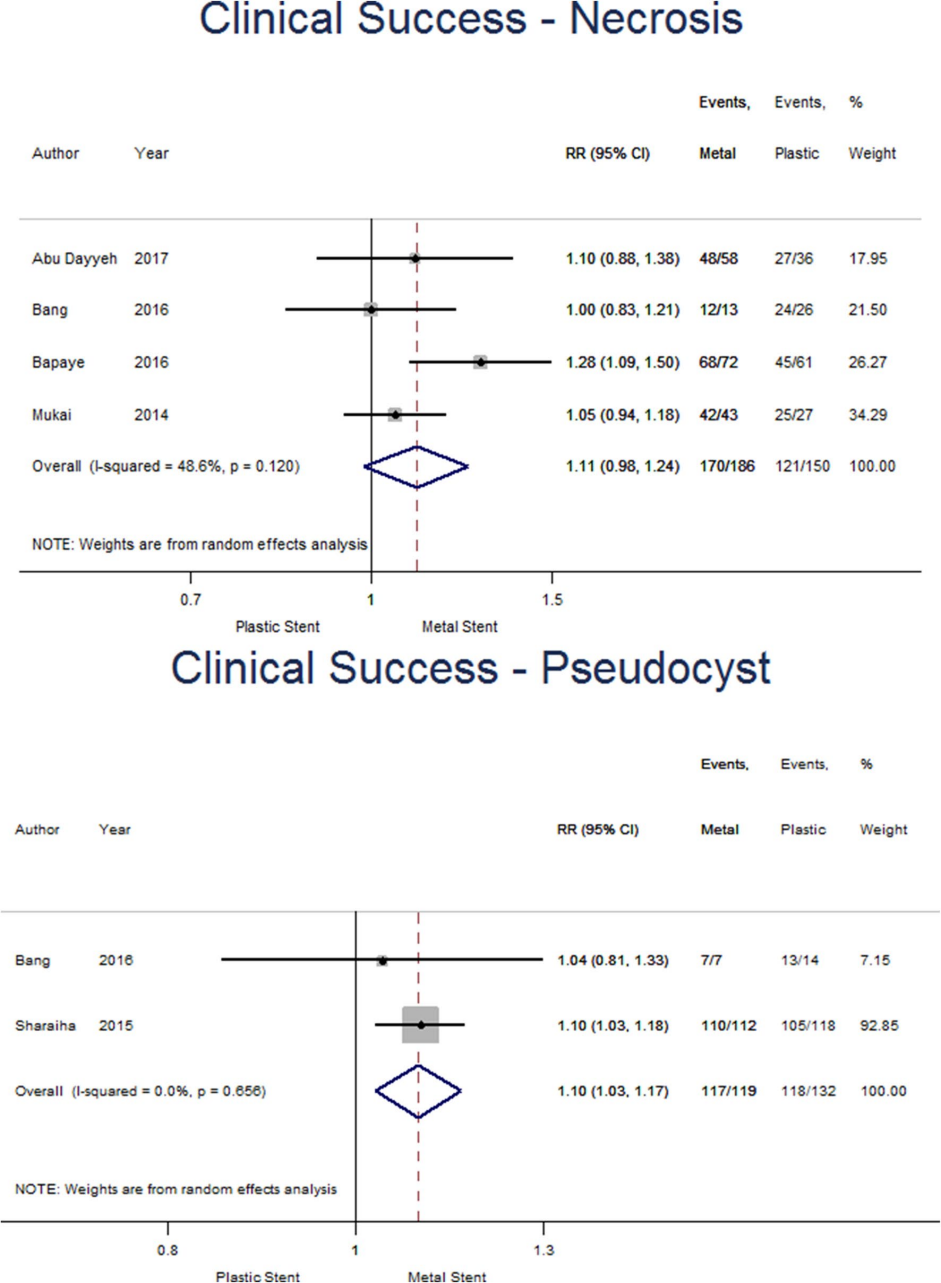
Forest plot showing individual and pooled risk ratios of clinical success for pseudocysts and walled-off necrosis

Subgroup analysis was undertaken and found four studies specific for WON, comprising 186 and 150 for metal and plastic stent groups, respectively (see Fig. 3). Only two studies were suitable for analysis for pseudocysts, including 119 patients with metal and 132 with plastic stents. For WON, clinical success was achieved in 91.4% of the metal stent group and 80.7% of patients with plastic stents. The pooled risk ratio suggests superiority of metal stents but does not reach significance (1.11 [95% CI 0.98–1.24], *p* = 0.089; *I*^2^ = 48.6%). Similarly, clinical success in the pseudocyst group occurred in 98.3% of those patients with metal stents and 89.4% of those with plastic stents. The pooled risk ratio (1.10 [95%CI 1.03–1.17], *p* = 0.005; *I*^2^ = 0.0%) suggests placing metal stents increases clinical success in patients with a pseudocyst, however, interpretation is limited due to the small number of studies included.

#### Adverse events

**Fig. 4:**
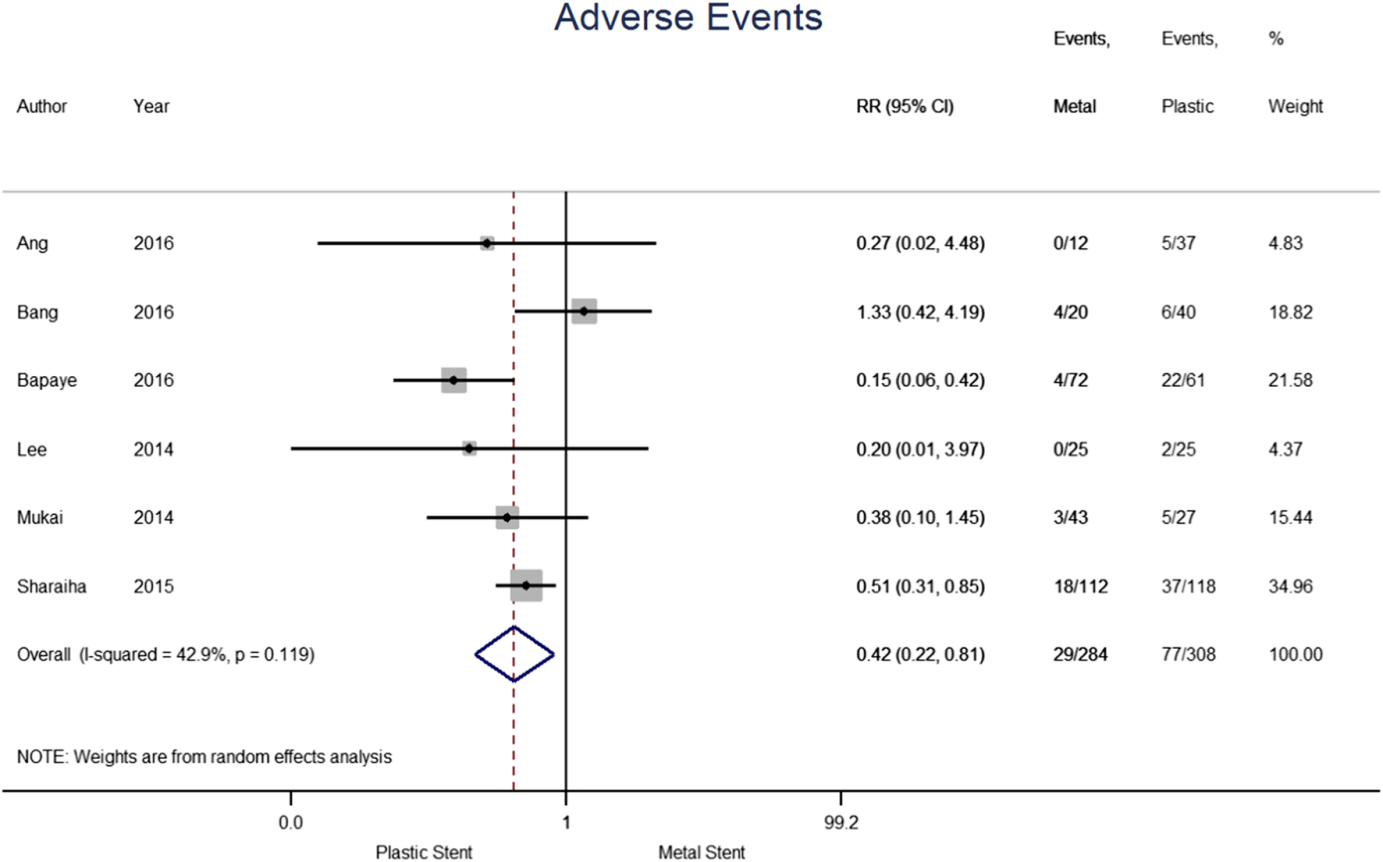
Forest plot for individual and pooled risk ratio of adverse events

The adverse events reported in individual studies are summarised in Table 5. A total of 592 patients from six studies were considered for this analysis; 284 in the metal and 308 in the plastic stent group (see Fig. 4). Adverse events were noted in 10.2% of the metal and 25.0% in plastic stent group. The pooled risk ratio demonstrated a 58% reduced risk of experiencing adverse events when a metal stent was used compared to plastic (0.42 [95% CI 0.22–0.81], *p* = 0.010; *I*^2^ = 42.9%). Results from Dayyeh et al. were not included as the summaries were reported for each adverse event separately. Random effects models for stent migration and perforation were fitted, however, no significant effect size between the two types of stents was identified.

**Table 5:** Frequency of specific adverse events

Author year	Bleeding (%)		Stent migration (%)		Infection (%)		Perforation (%)		Tract dilatation (%)	
	Plastic	Metal	Plastic	Metal	Plastic	Metal	Plastic	Metal	Plastic	metal
Ang et al. 2016 [24]	5.4	0.0	Cross-over of stent summary presented*		2.7	0.0	2.7	0.0	8.0	8.0
Bang et al. 2016 [19]	–		2.5	10.0	12.5	15.0	–		12.0–15.0	–
Bapaye et al. 2016 [20]	8.2	2.8	3.3	2.8	26.2	2.8	–		18.0	6.0
Dayyeh et al. 2017 [23]	19.4	6.9	19.4	20.7	5.6	3.4	8.3	1.7	15.0–18.0	15.0–18.0
Lee et al. 2014 [22]	4.0	0.0	4.0	0.0	8.0	12.0	–		8.0	When resistance encountered
Mukai et al. 2014 [21]	11.1	0.0	3.7	4.7	–		0.0	2.3	15.0–20.0	–
Sharaiha et al. 2015 [17]	5.1	2.7	0.8	0.9	13.6	5.4	4.2	1.8	10.0	10.0

*Ang et al. reports stent migration for stent cross-over

**Fig. 5:**
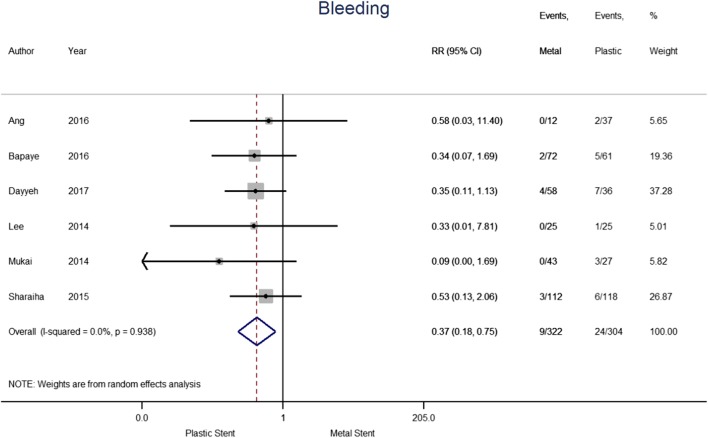
Forest plot for individual and pooled risk ratio of bleeding

The outcome of bleeding was analysed separately (see Fig. 5). The six papers included in the analysis contained a total of 626 patients, 322 of which treated with metal stents and 304 with plastic stents. Bleeding was reported for 2.8% and 7.9% of patients treated with metal and plastic stents, respectively. The pooled Risk Ratio indicates that the use of metal stents reduced the risk of bleeding by 63% compared to plastic stents (0.37; [95% CI 0.18–0.75], *p* = 0.006; *I*^2^ = 0.0%). The results do not show heterogeneity, suggesting bleeding risk was consistent across the publications. Results from Bang et al. were not included as it does not specifically report bleeding adverse events.

**Fig. 6:**
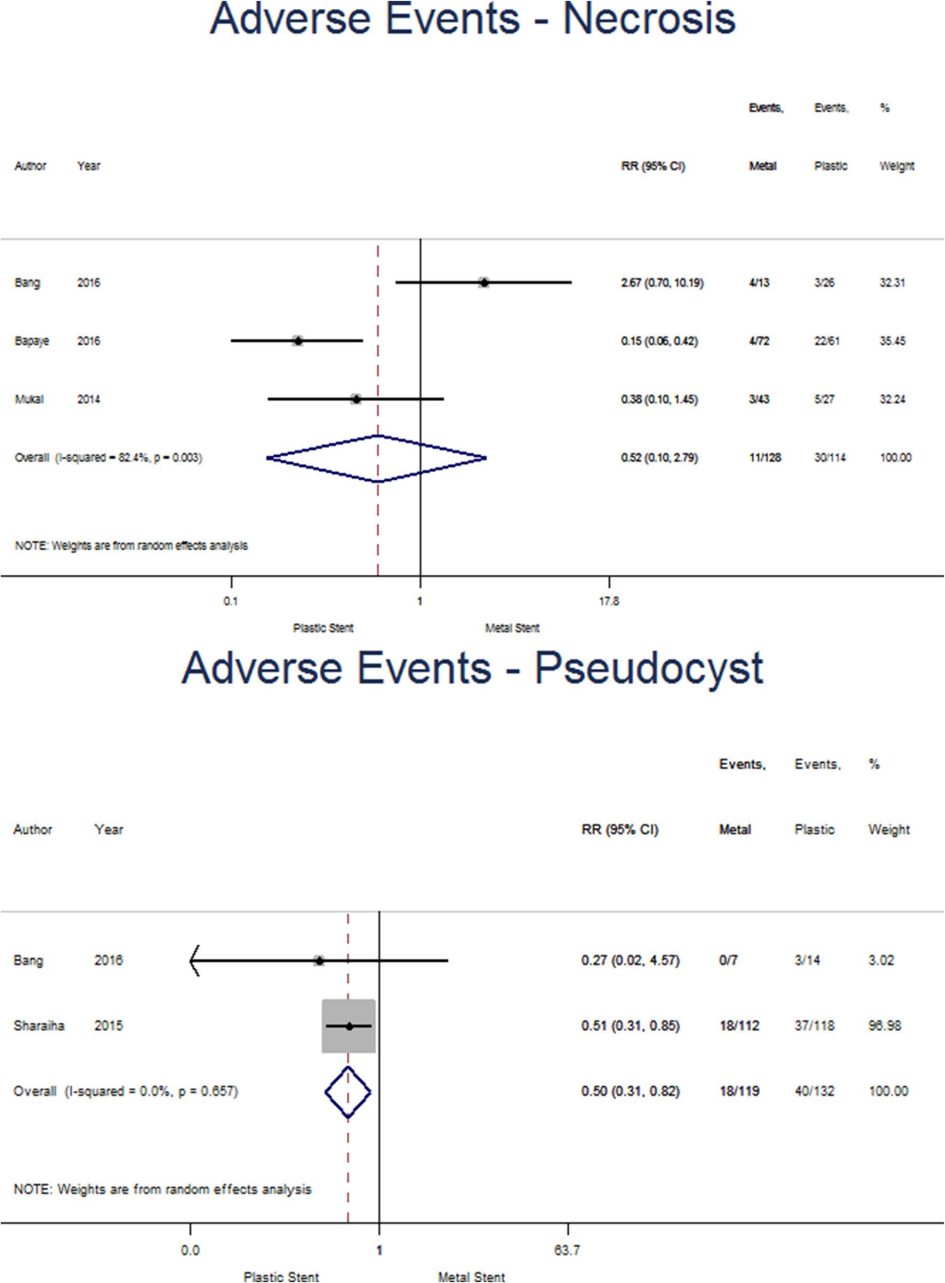
Forest plot showing individual and pooled risk ratios for adverse events for pseudocysts and walled-off necrosis

Subgroup analysis was undertaken and found three studies reporting adverse events for WON separately and were included in the analysis, 128 patients had metal stents included and 114 for plastic (see Fig. 6). Adverse events occurred in 8.6% of patients with metal stents and 26.3% of the plastic stent group. The pooled risk ratio (0.52 [95%CI 0.10–2.79], *p* = 0.442; *I*^2^ = 82.4%) does not suggest a significant reduction in adverse events for either plastic or metal stents for patients with WON. Two studies with 119 and 132 patients with metal and plastic stents, respectively, were likewise reported for pseudocysts. Adverse events occurred in 15.1% of patients with metal stents and 30.3% of those with plastic stents. The pooled risk ratio (0.50 [0.31–0.82], *p* = 0.006; *I*^2^ = 0.0%) suggests that inserting a metal stent reduced the risk of experiencing an adverse event in patients with pseudocysts.

The infection rate post stent insertion for metal stents was 5.4% and 13.2% for plastic stents. The pooled risk ratio (0.53 [95% CI 0.23–1.20], *p* = 0.127; *I*^2^ = 41.9%) does not suggest a difference between the groups. The severity of post-procedural infection was not well defined within the studies [17, 24]. One study reported a single mortality from uncontrolled sepsis [21], another reported 2/58 (3%) of metal 2/36 and (6%) of plastic stent patients required transfer to intensive care for sepsis management [23]. In three studies, either surgical or endoscopic intervention was required for control of infection [19, 20, 22]. Bang et al. stated 3/20 (15%) and 5/40 (12.5%) patients in the metal and plastic groups, respectively, developed post-procedural infection, four patients were managed with further endoscopic procedures and three by surgical techniques but this is not specified by stent type [19]. Bapaye et al. reported 2/72 (2.8%) patients with metal and 16/61 (26.2%) with plastic stents developed infection that were all managed surgically [20]. In the study by Lee, 2/25 (8%) of metal and 3/25 (12%) of the plastic group were found to have post-procedural infection and were all managed with further endoscopic drainage [22].

#### Reintervention

**Fig. 7:**
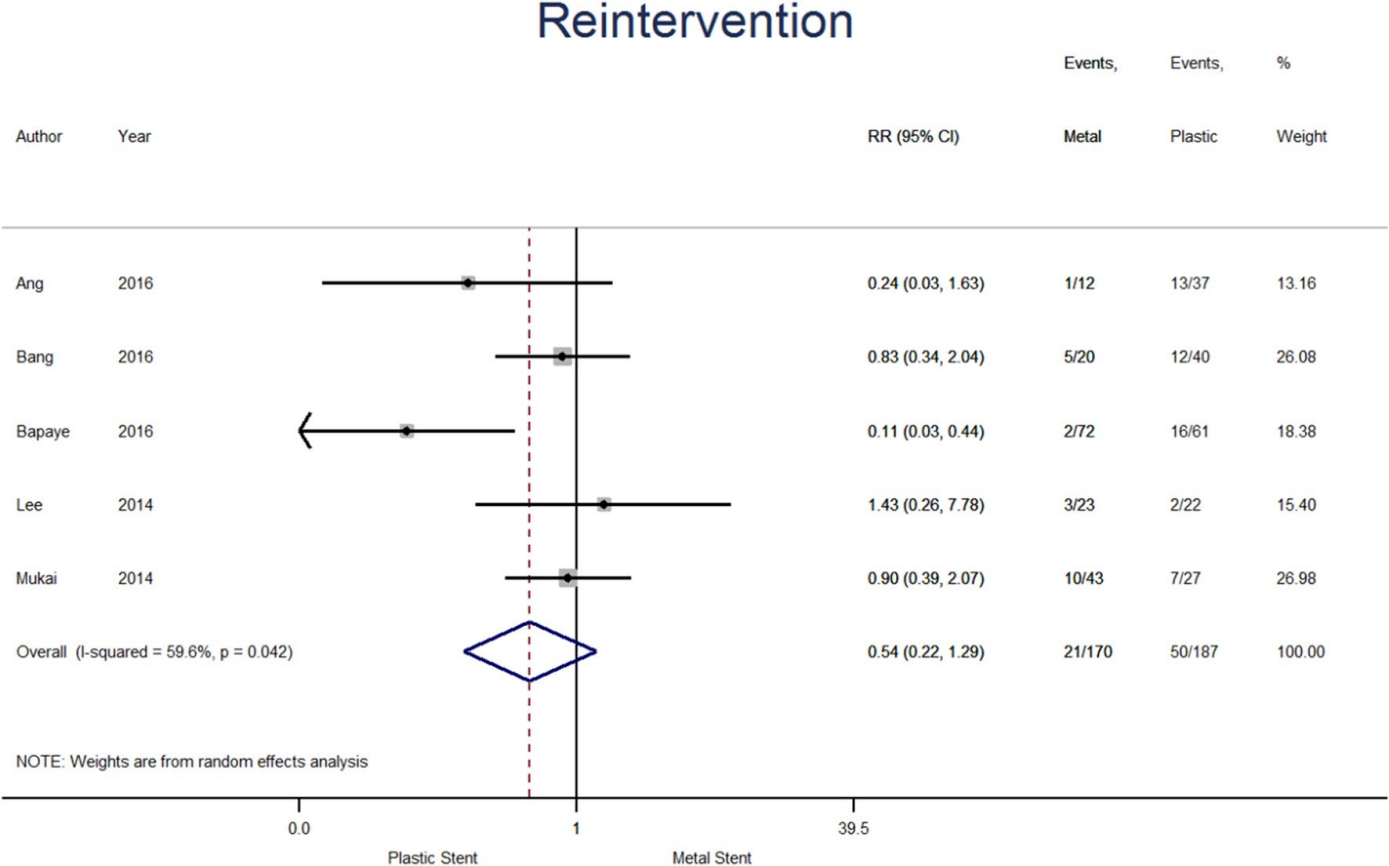
Forest plot for individual and pooled risk ratio for reintervention

Reintervention data were available from five studies (see Fig. 7), therefore the analysis contains 357 patients, 170 and 187 in metal and plastic stent groups, respectively. The percentage of patients requiring reintervention was 12.4% among those treated with metal stent and 26.7% in the plastic stent group. The pooled risk ratio suggests a higher risk of reintervention when plastic stent were used, however, treatment effect failed to reach statistical significance (0.54; [95% CI 0.22–1.29], *p* = 0.165; *I*^2^ = 59.6%).

The stated definitions for reintervention were a need for repeat endoscopy or surgery due to persistent symptoms associated with residual PFC that had not reduced by > 50% in size [24], if symptoms or inflammation continued despite drainage and additional sessions of direct endoscopic necrosectomy [21], additional transmural drainage and/or endoscopic necrosectomy [19] and salvage surgical intervention [20].

Sharaiha et al. and Dayyeh et al. were not included as reintervention rates were not reported fully. Sharaiha et al. stated that 52 (22%) patients required further interventions for pseudocysts within first month. Furthermore, it reported a significant difference in short-term intervention (*p* = 0.008) but does not include actual numbers or clarify which stent was superior [17].

**Fig. 8:**
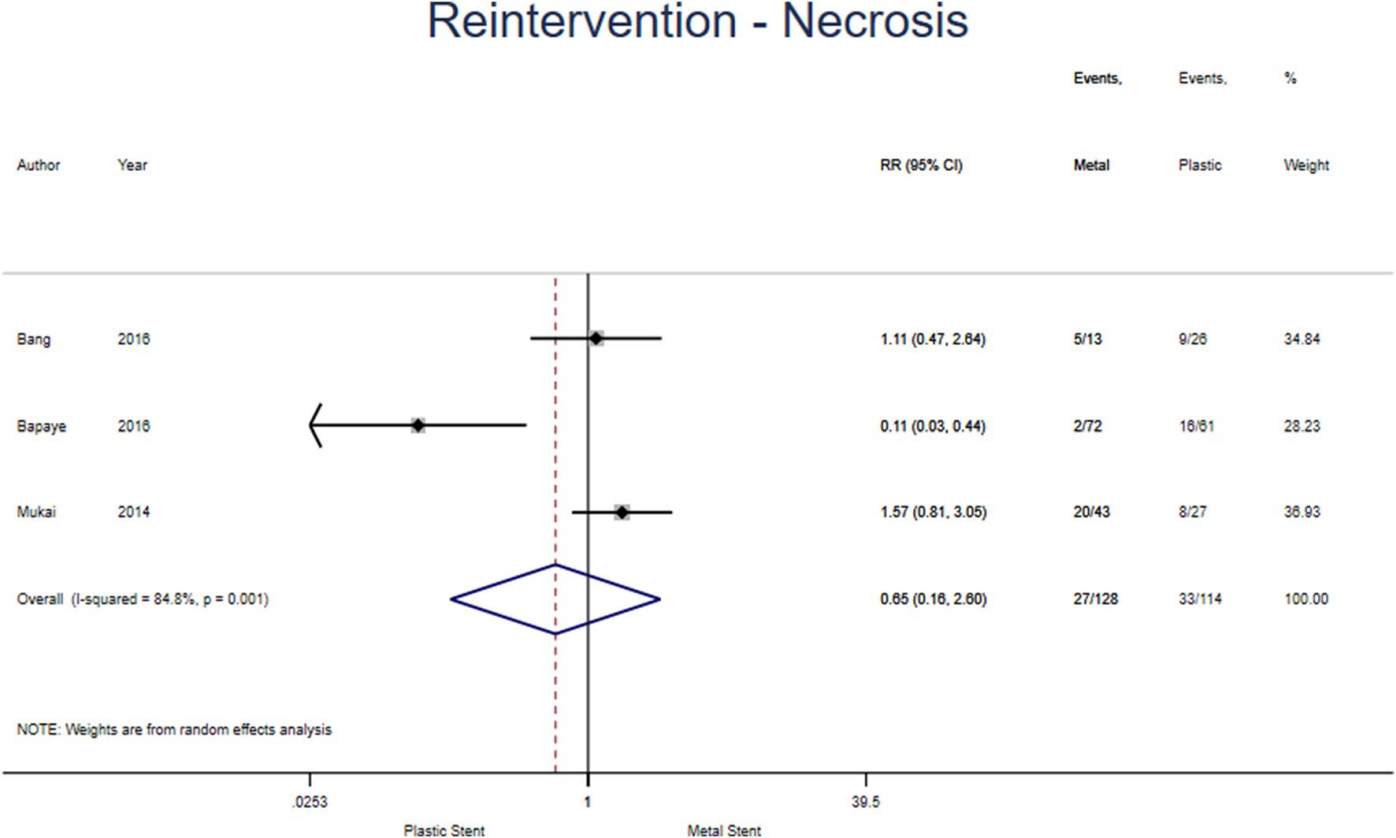
Forest plot showing individual and pooled risk ratios for reintervention in walled-off necrosis

Subgroup analysis was undertaken and found three studies specified reintervention in WON (see Fig. 8). 128 and 114 patients had metal and plastic stents inserted, respectively. 21.1% of those in the metal group and 22.9% in the plastic group required reintervention. The pooled risk ratio (0.65 [95% CI 0.16–2.60], *p* = 0.543; *I*^2^ = 84.8%) does not suggest a superiority for either stent. Only one study was suitable for inclusion in the pseudocyst analysis so meta-analysis was unable to be performed.

### Publication bias, subgroup and sensitivity analyses

The Bapaye study was a consistent outlier in the quantitative analysis. Sensitivity analyses performed without this study, confirmed the same findings of the main analyses and showed a considerable drop in heterogeneity. There was no significant difference in methodology or reporting to explain this and no reason to exclude it from the analysis.

Funnel plots to assess publication bias for outcomes are presented in supplementary information. The graphs do not reflect any publication bias and Egger’s regression tests for asymmetry yielded statistically non-significant p-values.

## Discussion

This meta-analysis demonstrates superior clinical success and reduced adverse events for use of metal stents when compared to plastic for endoscopic transluminal drainage of pancreatic fluid collections. Previous meta-analysis by Bang et al. showed no difference in the efficacy and adverse events between plastic and metal stents for drainage of PFCs [15]. The majority of these data were derived from the use of specifically designed, large calibre, covered metal stents with lumen apposing flanges, unlike the previous review. It is likely that the improved outcomes of metal stents in this review are as a result of these stents as they are tailored for PFC drainage.

The fistula created by balloon dilatation enables plastic stent placement and drainage of fluid, however, this may be insufficient due to spontaneous closure of the fistula around the stent. Plastic stents have substantially smaller lumens than metal stents leaving them more susceptible to blockage or occlusion, even in pseudocysts or WON with minimal debris. Although the use of plastic or metal stents was not found to reduce infection post drainage, metal stents can facilitate drainage of both liquid and the viscous necrotic debris, leading to the higher rates of successful drainage. Patients are not always routinely investigated by EUS prior to intervention; this is reflected in these studies where PFC’s were frequently diagnosed by CT or MR imaging. CT imaging has a low sensitivity for assessing necrosis so there is diagnostic uncertainty when judging a collection to be a pseudocyst or WON. Recent guidance suggests that MRI or ultrasound assessment may be required to accurately characterise the collection [3].

There are several limitations of this systematic review and meta-analysis. All but one included studies are retrospective studies with the inherent bias associated with this methodology [17, 19–23]. There was a discrepancy in type and quality of included studies leading to the synthesis of results of variable reliability [25, 26]. There were also differences between definitions for the outcomes reported. Different types of metal stent were used both in individual and across studies; it is not currently clear in published literature if there is any demonstrable clinical advantage of a particular stent. In two studies there is a discrete time point where practice changed and metal stents were used routinely; however, in four studies, plastic stents continued to be used for PFCs with certain characteristics leading to conceivable selection bias [21]. Furthermore, the sample sizes of some studies are relatively small and correspond to extended periods of time. The number of studies included in the meta-analyses is also quite limited and therefore meta-regression was not performed for exploring further the cause of heterogeneity.

All these studies were designed to investigate a difference in outcomes between stents not between types of PFC. There are limitations in combining pseudocysts and WON for data analysis and potential limitations in the classification of PFC within individual studies. The revised Atlanta criteria were introduced in 2012, therefore it is likely that patients were classified differently over the period the studies were ongoing. However, in the studies included except for Mukai et al. [21], patients have short or no length of stay recorded and no clinical details suggesting these are not acutely unwell patients with infected pancreatic necrosis but rather patients being treated on a semi-elective basis. The subgroup analyses for drainage of pseudocysts in terms of clinical success and adverse events suggest that metal stents remain advantageous over that of plastic stents. Similar subgroup analyses for drainage of WON with metal stents are less convincing, with clinical success almost reaching significance, while adverse events or reintervention show no difference between metal or plastic stents. However, these subgroup analyses are limited by the very small numbers of studies which state these indications separately and therefore it is difficult to draw conclusions based on these data. Finally, the cost incurred was not evaluated in this analysis.

Our analysis showed that patients were 58% less likely to experience an adverse event with metal compared to plastic stents. Inserting plastic stents, particularly multiple plastic stents can be technically demanding and time consuming which may in part explain the increased risk [22]. Bleeding was significantly more common in patients with a plastic stent (2.8 vs. 7.9%, *p* = 0.006), this may be due to the greater dilatation required for plastic stent insertion. Dilatation of the tract prior to stent insertion for plastic stents ranged from 8 to 20 mm and 0–18 mm for metal stents in studies included in this review. The majority of studies used multiple plastic stents inserted, which has previously been shown to improve treatment success compared to using a single stent [27].

Delayed bleeding in patients with metal stents was reported in one study [20]. An interim analysis for a randomised control trial by Bang et al. also reported significant delayed bleeding in 3 of 12 patients with LAMS [14]. This required a change in the trial protocol to remove stents earlier than initially planned. Investigators described buried stent syndrome in 2/12 patients and 1/12 patient with a biliary stricture secondary to a stent [14]. However, high rates of adverse events have not been seen in other cohorts of patients with LAMS [28]. The experience with LAMS is still early and more multicentre, prospective randomised data are required to accurately quantity the risk; elucidate causes for the risk and suggest potential solutions. It is likely that the delayed bleeding and buried stent problems seen with LAMS are due to its design rather than procedural steps in stent insertion.

There was no significant difference in the rate of reintervention between plastic and metal stents. This could be due to type 2 error as two of the largest studies were not included in the analysis and it was a relatively rare event for the sample size. Reported reintervention rates ranged from 2.8 to 35.1% between studies. The type of reintervention required also varied and was not always specified by authors. Bapaye et al. stated that salvage surgery was required in 26.2% of patients with plastic stents; however, Mukai et al. reported no patients required surgical intervention for inadequate drainage. This may suggest heterogeneity between included patients or difference in practice between centres.

EUS-guided drainage is regarded as first-line treatment for pancreatic fluid collections requiring intervention. The use of transmural metal stents increases the probability of clinical success and reduces the frequency of adverse events when compared to plastic stents for EUS-guided drainage of pancreatic fluid collections. Future, well-designed prospective randomised control trials with multiple centres are required to evaluate clinical outcomes, adverse events and potential costs.

## Electronic supplementary material

Below is the link to the electronic supplementary material.

Supplementary material 1 (DOCX 474 KB)

## References

[1] Imrie CW, Buist LJ, Shearer MG (1988). Importance of cause in the outcome of pancreatic pseudocysts. Am J Surg.

[2] Tyberg A, Karia K, Gabr M, Desai A, Doshi R, Gaidhane M, Sharaiha RZ, Kahaleh M (2016). Management of pancreatic fluid collections: a comprehensive review of the literature. World J Gastroenterol.

[3] Banks PA, Bollen TL, Dervenis C, Gooszen HG, Johnson CD, Sarr MG, Tsiotos GG, Vege SS (2013). Classification of acute pancreatitis-2012: revision of the Atlanta classification and definitions by international consensus. Gut.

[4] Neoptolemos JP, London NJ, Carr-Locke DL (1993). Assessment of main pancreatic duct integrity by endoscopic retrograde pancreatography in patients with acute pancreatitis. Br J Surg.

[5] Guidelines WGIAAP (2013). IAP/APA evidence-based guidelines for the management of acute pancreatitis. Pancreatology.

[6] van Santvoort HC, Besselink MG, Bakker OJ, Hofker HS, Boermeester MA, Dejong CH, van Goor H, Schaapherder AF, van Eijck CH, Bollen TL, van Ramshorst B, Nieuwenhuijs VB, Timmer R, Lameris JS, Kruyt PM, Manusama ER, van der Harst E, van der Schelling GP, Karsten T, Hesselink EJ, van Laarhoven CJ, Rosman C, Bosscha K, de Wit RJ, Houdijk AP, van Leeuwen MS, Buskens E, Gooszen HG (2010). A step-up approach or open necrosectomy for necrotizing pancreatitis. N Engl J Med.

[7] van Brunschot S, Hollemans RA, Bakker OJ, Besselink MG, Baron TH, Beger HG, Boermeester MA, Bollen TL, Bruno MJ, Carter R, French JJ, Coelho D, Dahl B, Dijkgraaf MG, Doctor N, Fagenholz PJ, Farkas G, Castillo CFD, Fockens P, Freeman ML, Gardner TB, Goor HV, Gooszen HG, Hannink G, Lochan R, McKay CJ, Neoptolemos JP, Olah A, Parks RW, Peev MP, Raraty M, Rau B, Rosch T, Rovers M, Seifert H, Siriwardena AK, Horvath KD, van Santvoort HC (2017). Minimally invasive and endoscopic versus open necrosectomy for necrotising pancreatitis: a pooled analysis of individual data for 1980 patients. Gut.

[8] Gomatos IP, Halloran CM, Ghaneh P, Raraty MG, Polydoros F, Evans JC, Smart HL, Yagati-Satchidanand R, Garry JM, Whelan PA, Hughes FE, Sutton R, Neoptolemos JP (2016). Outcomes from minimal access retroperitoneal and open pancreatic necrosectomy in 394 patients with necrotizing pancreatitis. Ann Surg.

[9] Varadarajulu S, Bang JY, Sutton BS, Trevino JM, Christein JD, Wilcox CM (2013). Equal efficacy of endoscopic and surgical cystogastrostomy for pancreatic pseudocyst drainage in a randomized trial. Gastroenterology.

[10] Vilmann AS, Menachery J, Tang SJ, Srinivasan I, Vilmann P (2015). Endosonography guided management of pancreatic fluid collections. World J Gastroenterol.

[11] Bakker OJ, van Santvoort HC, van Brunschot S, Geskus RB, Besselink MG, Bollen TL, van Eijck CH, Fockens P, Hazebroek EJ, Nijmeijer RM, Poley JW, van Ramshorst B, Vleggaar FP, Boermeester MA, Gooszen HG, Weusten BL, Timmer R (2012). Endoscopic transgastric vs surgical necrosectomy for infected necrotizing pancreatitis: a randomized trial. JAMA.

[12] Baron TH, Thaggard WG, Morgan DE, Stanley RJ (1996). Endoscopic therapy for organized pancreatic necrosis. Gastroenterology.

[13] Yamamoto N, Isayama H, Kawakami H, Sasahira N, Hamada T, Ito Y, Takahara N, Uchino R, Miyabayashi K, Mizuno S, Kogure H, Sasaki T, Nakai Y, Kuwatani M, Hirano K, Tada M, Koike K (2013). Preliminary report on a new, fully covered, metal stent designed for the treatment of pancreatic fluid collections. Gastrointest Endosc.

[14] Bang JY, Hasan M, Navaneethan U, Hawes R, Varadarajulu S (2016). Lumen-apposing metal stents (LAMS) for pancreatic fluid collection (PFC) drainage: may not be business as usual. Gut.

[15] Bang JY, Hawes R, Bartolucci A, Varadarajulu S (2015). Efficacy of metal and plastic stents for transmural drainage of pancreatic fluid collections: a systematic review. Dig Endosc.

[16] Moher D, Liberati A, Tetzlaff J, Altman DG (2009). Preferred reporting items for systematic reviews and meta-analyses: the PRISMA statement. BMJ.

[17] Sharaiha RZ, DeFilippis EM, Kedia P, Gaidhane M, Boumitri C, Lim HW, Han E, Singh H, Ghumman SS, Kowalski T, Loren D, Kahaleh M, Siddiqui A (2015). Metal versus plastic for pancreatic pseudocyst drainage: clinical outcomes and success. Gastrointest Endosc.

[18] Siddiqui AA, Kowalski TE, Loren DE, Khalid A, Soomro A, Mazhar SM, Isby L, Kahaleh M, Karia K, Yoo J, Ofosu A, Ng B, Sharaiha RZ (2016). Fully covered self-expanding metal stents versus lumen-apposing fully covered self-expanding metal stent versus plastic stents for endoscopic drainage of pancreatic walled-off necrosis: clinical outcomes and success. Gastrointest Endosc.

[19] Bang JY, Hasan MK, Navaneethan U, Sutton B, Frandah W, Siddique S, Hawes RH, Varadarajulu S (2016). Lumen apposing metal stents (LAMS) for drainage of pancreatic fluid collections: when and for whom?. Dig Endosc.

[20] Bapaye A, Dubale NA, Sheth KA, Bapaye J, Ramesh J, Gadhikar H, Mahajani S, Date S, Pujari R, Gaadhe R (2016). Endoscopic ultrasonography-guided transmural drainage of walled-off pancreatic necrosis: comparison between a specially designed fully covered bi-flanged metal stent and multiple plastic stents. Dig Endosc.

[21] Mukai S, Itoi T, Baron TH, Sofuni A, Itokawa F, Kurihara T, Tsuchiya T, Ishii K, Tsuji S, Ikeuchi N, Tanaka R, Umeda J, Tonozuka R, Honjo M, Gotoda T, Moriyasu F, Yasuda I (2015). Endoscopic ultrasound-guided placement of plastic vs. biflanged metal stents for therapy of walled-off necrosis: a retrospective single-center series. Endoscopy.

[22] Lee BU, Song TJ, Lee SS, Park DH, Seo DW, Lee SK, Kim MH (2014). Newly designed, fully covered metal stents for endoscopic ultrasound (EUS)-guided transmural drainage of peripancreatic fluid collections: a prospective randomized study. Endoscopy.

[23] Abu Dayyeh BK, Mukewar S, Majumder S, Zaghlol R, Vargas Valls EJ, Bazerbachi F, Levy MJ, Baron TH, Gostout CJ, Petersen BT, Martin J, Gleeson FC, Pearson RK, Chari ST, Vege SS, Topazian MD (2017). Large-caliber metal stents versus plastic stents for the management of pancreatic walled-off necrosis. Gastrointest Endosc.

[24] Ang TL, Kongkam P, Kwek AB, Orkoonsawat P, Rerknimitr R, Fock KM (2016). A two-center comparative study of plastic and lumen-apposing large diameter self-expandable metallic stents in endoscopic ultrasound-guided drainage of pancreatic fluid collections. Endosc Ultrasound.

[25] Higgins J, Green S (eds) (2011) Cochrane handbook for systematic reviews of interventions version 5.1.0 (updated March 2011). The Cochrane Collaboration. http://handbook.cochrane.org. Accessed 5 Sept 2018

[26] Wells GA SB, O’Connell D, Peterson J, Welch V, Losos M, Tugwell P (2017) The Newcastle-Ottawa Scale (NOS) for assessing the quality of nonrandomised studies in meta-analyses. http://www.ohri.ca/programs/clinical_epidemiology/oxford.htm. Accessed 20 Oct 2017

[27] Bang JY, Wilcox CM, Trevino J, Ramesh J, Peter S, Hasan M, Hawes RH, Varadarajulu S (2013). Factors impacting treatment outcomes in the endoscopic management of walled-off pancreatic necrosis. J Gastroenterol Hepatol.

[28] Ryan BM, Venkatachalapathy SV, Huggett MT (2016). Safety of lumen-apposing metal stents (LAMS) for pancreatic fluid collection drainage. Gut.

