# Supplementary material for: A systematic review and meta-analysis of metal versus plastic stents for drainage of pancreatic fluid collections: metal stents are advantageous
Source: Surg Endosc. 2018 Sep 6;33(5):1412–25. doi: 10.1007/s00464-018-6416-5 (PMC6484810; doi:10.1007/s00464-018-6416-5)

**SUPPLENTARY INFORMATION**

**Quality of included studies**

Table s1: Newcastle-Ottawa Quality assessment of included studies [26]

| **Study** | **Study Design** | **Selection** | **Comparability** | **Outcome** | **Quality assessment** |
| --- | --- | --- | --- | --- | --- |
| Ang et al  2016 [24] | Retrospective  2 centre | **** | * | * | Poor |
| Bang et al 2016 [19] | Retrospective  case control | **** | ** | *** | Good |
| Bapaye et al  2016 [20] | Retrospective | **** | ** | ** | Good |
| Dayyeh et al 2017 [23] | Retrospective | **** | ** | * | Poor |
| Mukai et al 2014 [21] | Retrospective | **** | ** | *** | Good |
| Sharaiha et al 2015 [17] | Retrospective  2 centre cohort | **** | ** | *** | Good |

Table s2: Cochrane risk of bias table [25]

| *Lee et al [22]* |  |  |
| --- | --- | --- |
| **Bias** | **Authors' judgement** | **Support for judgement** |
| Random sequence generation (selection bias) |  | Not stratified. |
| Allocation concealment (selection bias) |  | Allocation revealed just before cyst puncture which is the latest possible time. |
| Blinding of participants and personnel (performance bias) |  | Methods for blinding to patients not described. Patients only under sedation. Single blinded trial |
| Blinding of outcome assessment (detection bias) |  | Single blinded so clinicians at follow up would know which stent was used. However outcome measures are objective and at low risk of bias. |
| Incomplete outcome data (attrition bias) |  | 10% patients lost to follow up |
| Selective reporting (reporting bias) |  | All outcomes stated reported |
| Other bias |  |  |

Figure s1: Funnel plot for clinical success


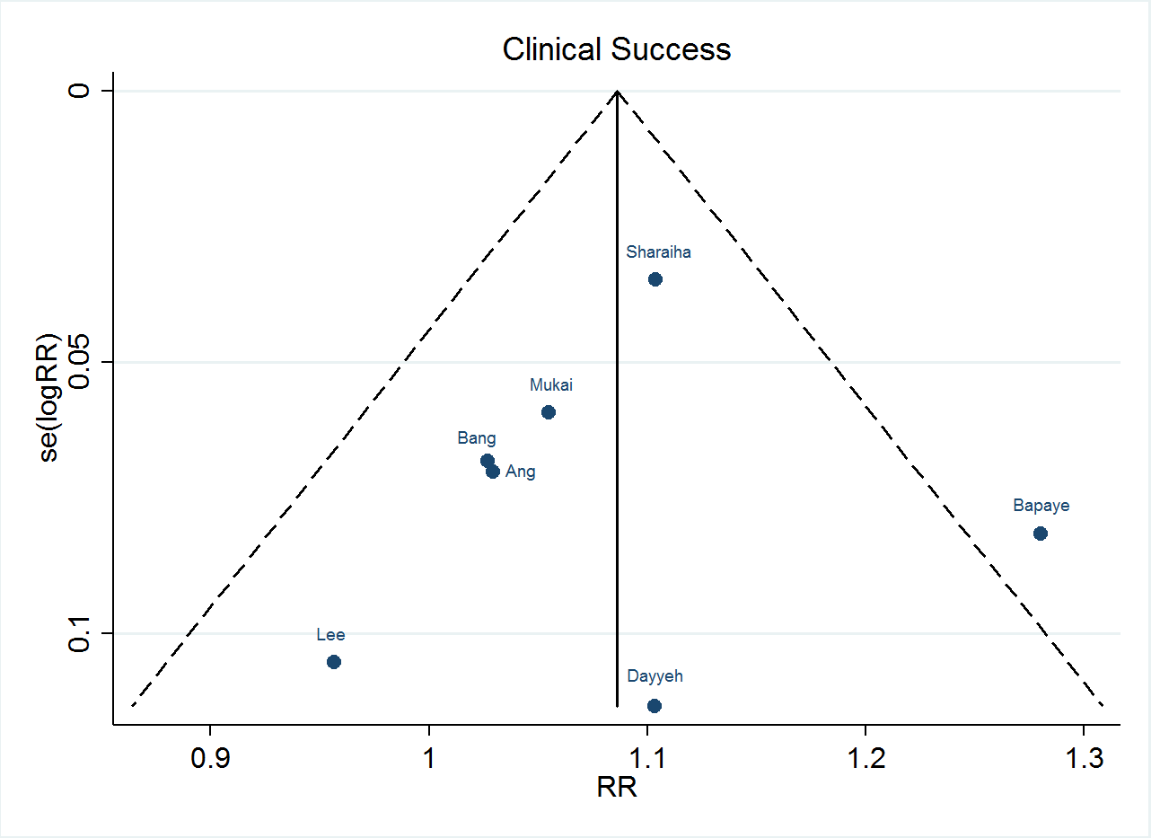


Figure s2: Funnel plot for adverse events


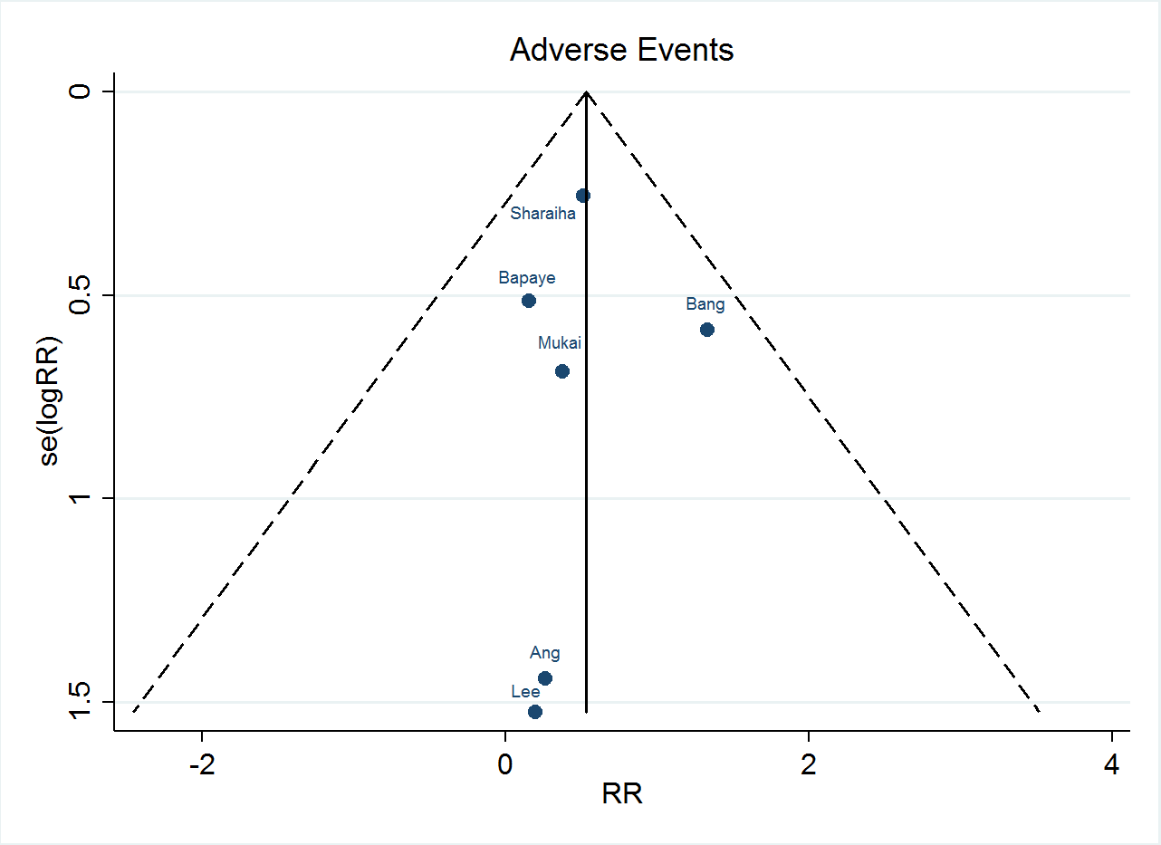


Figure s3: Funnel plot for bleeding


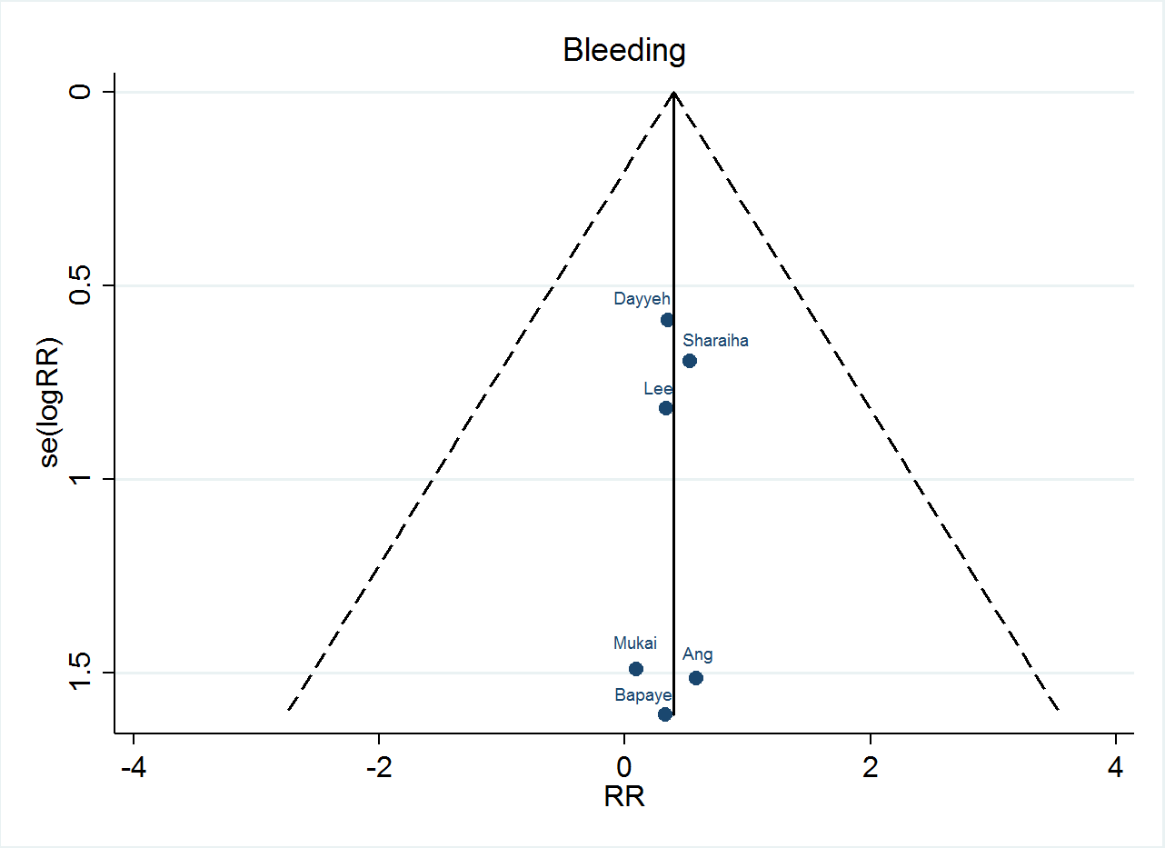


Figure s4: Funnel plot for reintervention


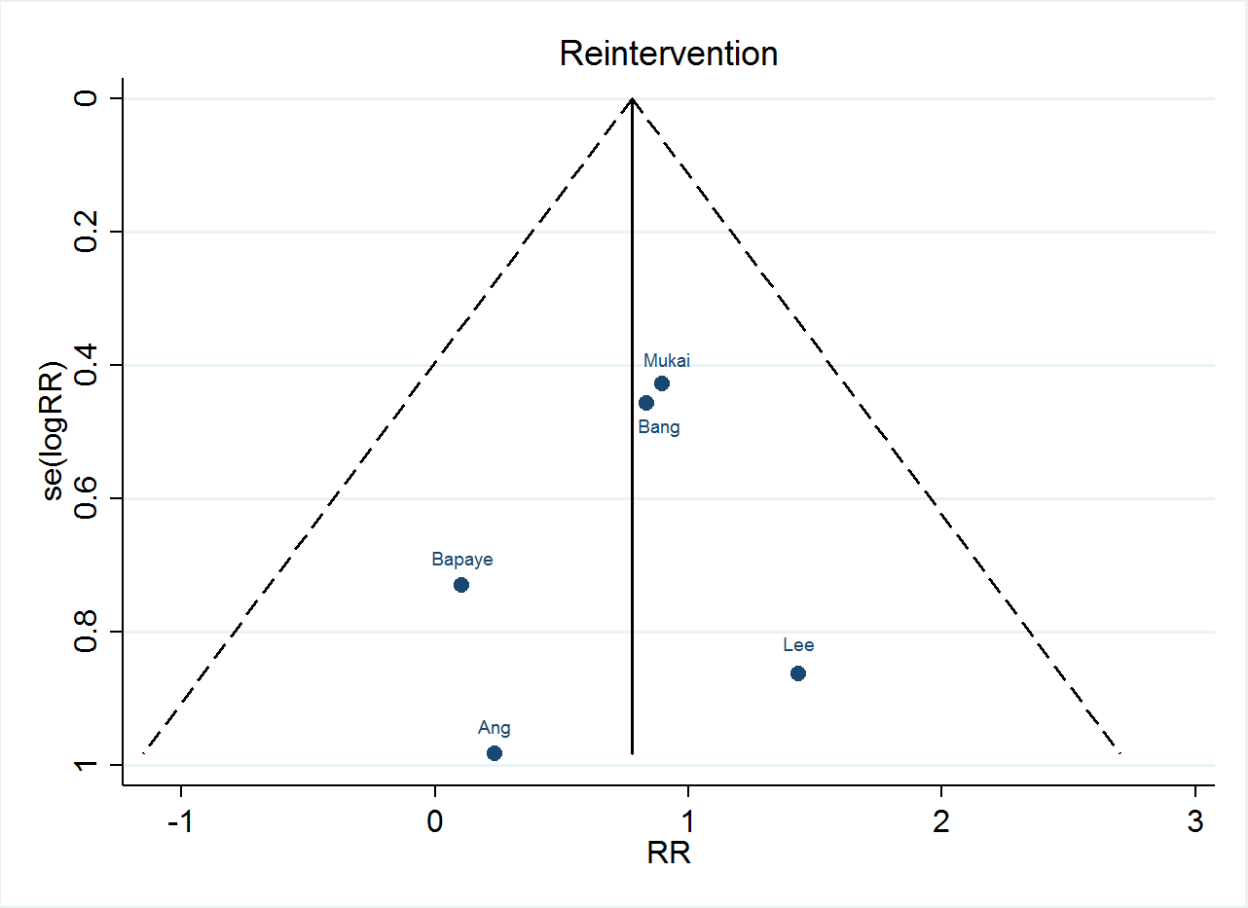

Supplement: Supplementary file 1 — Supplementary material 1 (DOCX 474 KB) [file 464_2018_6416_MOESM1_ESM.docx]
